# Acquiring and maintaining a normal oral microbiome: current perspective

**DOI:** 10.3389/fcimb.2014.00085

**Published:** 2014-06-26

**Authors:** Egija Zaura, Elena A. Nicu, Bastiaan P. Krom, Bart J. F. Keijser

**Affiliations:** ^1^Department of Preventive Dentistry, Academic Centre for Dentistry AmsterdamAmsterdam, Netherlands; ^2^Department of Periodontology, Academic Centre for Dentistry AmsterdamAmsterdam, Netherlands; ^3^Microbiology and Systems Biology, TNO Earth, Environmental and Life SciencesZeist, Netherlands; ^4^Top Institute Food and NutritionWageningen, Netherlands

**Keywords:** oral microbiome, placenta, tolerance, mucosal immunity, stability, colonization resistance

## Abstract

The oral microbiota survives daily physical and chemical perturbations from the intake of food and personal hygiene measures, resulting in a long-term stable microbiome. Biological properties that confer stability in the microbiome are important for the prevention of dysbiosis—a microbial shift toward a disease, e.g., periodontitis or caries. Although processes that underlie oral diseases have been studied extensively, processes involved in maintaining of a normal, healthy microbiome are poorly understood. In this review we present our hypothesis on how a healthy oral microbiome is acquired and maintained. We introduce our view on the prenatal development of tolerance for the normal oral microbiome: we propose that development of fetal tolerance toward the microbiome of the mother during pregnancy is the major factor for a successful acquisition of a normal microbiome. We describe the processes that influence the establishment of such microbiome, followed by our perspective on the process of sustaining a healthy oral microbiome. We divide microbiome-maintenance factors into host-derived and microbe-derived, while focusing on the host. Finally, we highlight the need and directions for future research.

## Introduction

The oral microbiota needs to cope with daily physical and chemical perturbations from the intake of food and personal hygiene measures. These include fluctuations in temperature, pH, antimicrobial and dietary components, and mechanical sheer forces from brushing and mastication. Intriguingly, a long-term stable microbiome is maintained in the oral cavity, as demonstrated by Rasiah and colleagues by following an individual saliva donor over a period of 7 years (Rasiah et al., 2005). Recent data from the NIH Human Microbiome Project (HMP) revealed that the oral microbiome has the largest core of commonly shared microbes among unrelated individuals compared to other habitats such as gut or skin (Costello et al., 2009; Li et al., 2013; Zhou et al., 2013).

A key question is what governs the stability of the oral microbiome in health? Biological properties that confer stability in the microbiome are important for the prevention of dysbiosis—a microbial shift toward a disease, e.g., periodontitis or caries and sustaining general health (for review see Wade, 2013). Although processes that underlie oral diseases have been studied extensively (Bartold and Van Dyke, 2013; Bradshaw and Lynch, 2013; Nyvad et al., 2013; Belibasakis, 2014), processes behind the maintaining of a normal microbiome are poorly understood. In this review we present our hypothesis on how a healthy oral microbiome is acquired and maintained. We start by defining what constitutes a normal oral microbiome. Then we present our hypothesis on the mechanisms for acquiring a stable normal microbiome. Finally, we discuss some of the mechanisms involved in maintaining such a microbiome and highlight the directions for possible further research.

## What constitutes normal oral microbiome?

The human oral cavity is colonized by a wide range of microorganisms. Besides bacteria and fungi, Archaea, viruses and protozoa form a part of a normal microbiome (Wade, 2013). Current reports on a normal oral microbiome however are limited to the “bacteriome” (subsequently referred to as “microbiome”) and very limited reports on the mycobiome—fungal microbiome (Ghannoum et al., 2010; Dupuy et al., 2014; Mukherjee et al., 2014). Current knowledge on the role of fungi as part of a healthy oral microbiome has been recently reviewed and is therefore not further discussed here (Krom et al., 2014). The microbiome has been studied in great detail and phylogenetic information of oral bacteria is gathered in databases dedicated to oral cavity (Palmer, 2014). The HMP assessed microbiome composition of nine intraoral sites (buccal mucosa, hard palate, keratinized gingiva, palatine tonsils, saliva, sub- and supragingival plaque, throat and tongue dorsum) from about 200 subjects and found 185–355 genera, belonging to 13–19 bacterial phyla (Zhou et al., 2013). An individual sample (i.e., from a single site of a single volunteer) contained sequences classified to 20–50 genera from 6 to 9 phyla. Table 1 summarizes the high abundance core genera (defined as genera present at >10% abundance and at >75% ubiquity) and other major core genera (>1% abundance at >80% ubiquity), as well as operational taxonomic units (OTUs, 16S rRNA gene variable v3–v5 region sequences, clustered at 97% similarity) in these oral samples (Li et al., 2013). A single OTU, *Streptococcus* OTU#2, dominated nearly all oral mucosal sites of this large cohort. The reads of this OTU were obtained from Kelvin Li (personal communication) and blasted (NCBI web site, megablast against 16S ribosomal RNA sequences, default parameters) for finer taxonomic classification. The most abundant read of this OTU was identical over its entire length to 16S rRNA gene sequences of *Streptococcus oralis, Streptococcus mitis* and *Streptococcus peroris*.

**Table 1:** **The core bacterial taxa in the oral cavity from over 200 healthy individuals participating in HMP (Li et al., 2013)**.

**Sample type**	**High abundance core genera in >75% samples at >10% abundance**	**Other major core genera in >80% samples at >1% abundance**	**Minor core genera in >50% samples**
Buccal mucosa	*Streptococcus (2)*	*Uncl. Pasteurellaceae (16, 19)*	*Atopobium*
		*Gemella (11)*	*Uncl. Prevotellaceae*
			*Uncl. Bacilli*
			*Catonella*
Hard palate	*Streptococcus (2, 6)*	*Uncl. Pasteurellaceae (16)*	*Mogibacterium*
		*Veillonella (4)*	*Catonella*
		*Prevotella (10)*	
		*Uncl. Lactobacillales (13)*	
		*Gemella (11)*	
Keratinized gingiva	*Streptococcus (2)*		*Uncl. Bacilli*
	*Uncl. Pasteurellaceae (19)*		
Palatine tonsils		*Streptococcus (2, 6)*	*Mogibacterium*
		*Veillonella (4)*	*Uncl. Firmicutes*
		*Prevotella (10)*	
		*Fusobacterium (9)*	
		*Uncl. Pasteurellaceae (16)*	
Saliva		*Prevotella (10)*	*Uncl. Actinomycetales*
		*Streptococcus (2, 6)*	*Tannerella*
		*Veillonella (4)*	*Kingella*
		*Uncl. Pasteurellaceae (16)*	
		*Fusobacterium (9)*	
		*Porphyromonas (7)*	
		*Neisseria (−)*	
Subgingival plaque		*Streptococcus (2)*	*Uncl. Firmicutes*
		*Fusobacterium (9)*	
		*Capnocytophaga (−)*	
		*Prevotella (−)*	
		*Corynebacterium (−)*	
		*Uncl. Pasteurellaceae(−)*	
Supragingival plaque		*Streptococcus (2)*	*Uncl. Betaproteobacteria*
		*Capnocytophaga (−)*	
		*Corynebacterium (15)*	
		*Uncl. Pasteurellaceae (−)*	
		*Uncl. Neisseriaceae (21)*	
		*Fusobacterium (9)*	
Throat	*Streptococcus (2, 6)*	*Veillonella (4)*	*Mogibacterium*
		*Prevotella (10)*	*Uncl. Firmicutes*
		*Uncl. Pasteurellaceae (16)*	
		*Actinomyces (−)*	
		*Fusobacterium (9)*	
		*Uncl. Lachnospiraceae (−)*	
Tongue dorsum	*Streptococcus (2, 6)*	*Veillonella (4)*	*Uncl. Actinomycetales*
		*Prevotella (10)*	*Uncl. Bacilli*
		*Uncl. Pasteurellaceae (16)*	*Peptostreptococcus*
		*Actinomyces (14)*	
		*Fusobacterium (9)*	
		*Uncl. Lactobacillales (13)*	
		*Neisseria (8)*	

*In the parentheses—the corresponding OTU in the genus or family. Uncl, unclassified*.

**Figure 1:**
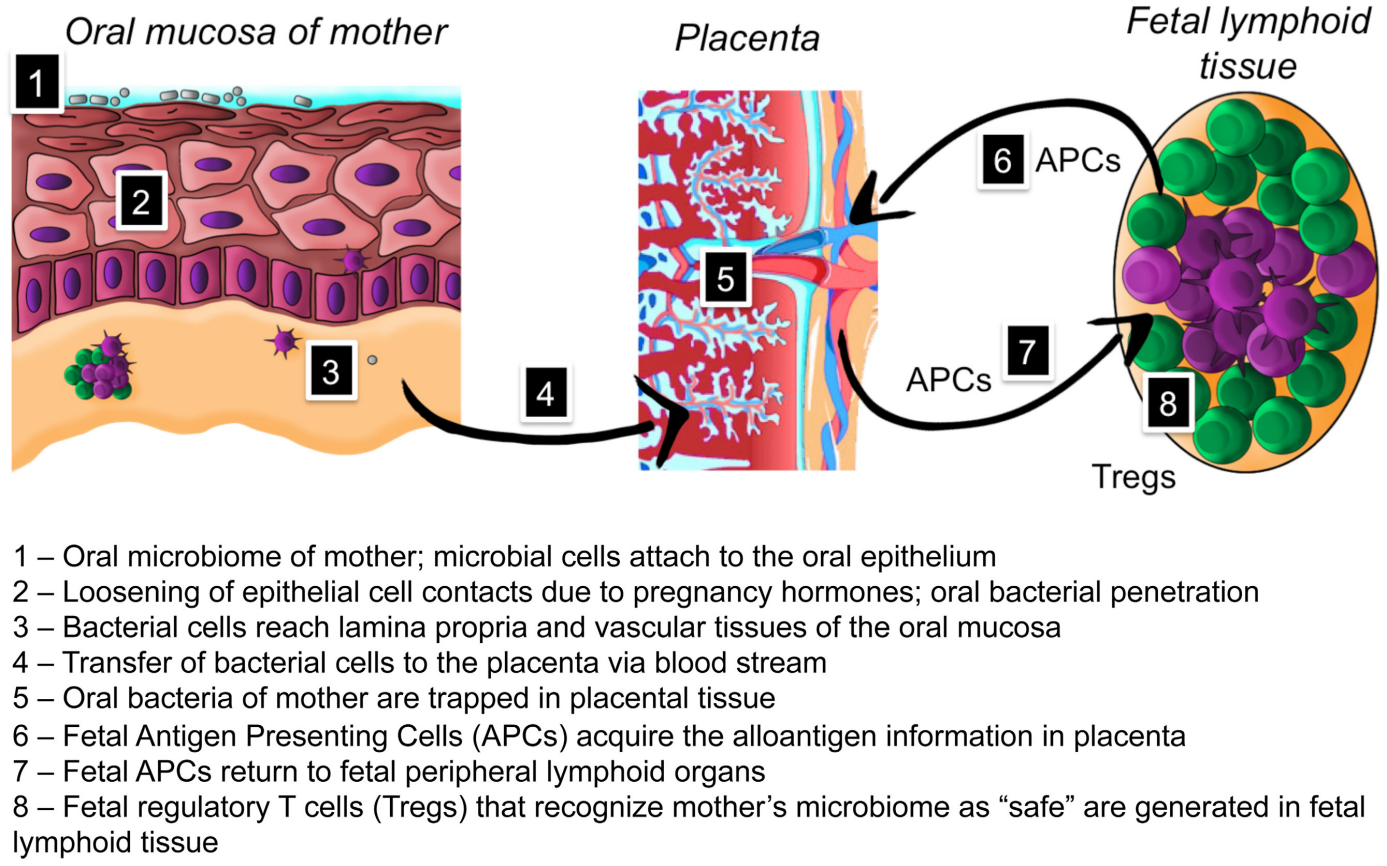
**Hypothesis on the role of placental microbiome in the development of fetal tolerance toward oral microbiome of mother**.

The microbiome has evolved through hundreds of thousands of years of co-habiting into a microbe-human symbiosis with mutual benefits (Hooper and Gordon, 2001; Clemente et al., 2012). The oral microbiome in newborns has been shown to seed the gut microbiome that first resembles that of the oral cavity and diverges in 2 weeks time to gut-specific communities (Costello et al., 2013). Recently a significant association between distinct microbial community types of stool and oral samples from HMP study adult population was demonstrated (Ding and Schloss, 2014). Based on current knowledge it is apparent that the acquisition of such normal, beneficiary microbiome by newborns is an essential process. Infants are colonized rapidly after birth by bacteria present in their direct environment, through bacterial transfer from their mother but also from other sources. How does a newborn discriminate between “friend and foe” among the diverse microbes in its postnatal environment? If any randomly trespassing microbe would permanently colonize oral cavity of the newborn, there would be no core microbiome (Table 1) and the benefit of the long-lasting co-evolution would be lost. We suggest that development of fetal tolerance toward the microbiome of the mother during pregnancy is the major factor for a successful acquisition of a normal microbiome. We have summarized our hypothesis in the text below and in Figure 1.

## Development of prenatal tolerance to mother's oral microbiome

Although the first encounter of a newborn with microbiota is considered to be postnatal, there is clinical evidence for microbial presence in placenta, umbilical cord blood, amniotic fluid, and meconium in full-term pregnancies without overt infection (Bearfield et al., 2002; Jiménez et al., 2005, 2008; Stout et al., 2013; Aagaard et al., 2014). Experimental intravenous infection of pregnant mice with pooled salivary or plaque microbes resulted in colonization of placenta by selected oral microorganisms (Fardini et al., 2010). Recent comparison of sequencing results from 320 placental microbiomes with the HMP dataset showed that the placental microbiome does not resemble vaginal or gut microbiomes as previously thought, but at least at a phylum level is most similar to normal oral microbiome, especially that from tongue and tonsils (Aagaard et al., 2014). These studies may suggest that the placental microbiome has a biological function. We propose that during pregnancy the placenta becomes an antigen-collecting site for the fetal immune system to be “trained” in antigen tolerance. We suggest a hematogenous route for indigenous microbes to placenta during pregnancy. Pregnant women have increased gingival bleeding, diagnosed as pregnancy gingivitis (Niederman, 2013). We propose a new role for the increased gingival bleeding: by opening the vascular bed, oral bacteria from the mother become available in blood and thus gain access to the placenta. Jeurink and colleagues have introduced a similar mechanism for the formation of the breast milk microbiome. This involves immune cell education by the pregnancy hormone progesterone resulting in transportation of bacteria from the mother to her mammal glands (Jeurink et al., 2013). A similar process could be responsible for transporting bacteria to the placenta. Microbial cells are trapped in placental tissue to be presented to the fetal immune system. In the prenatal period, fetal antigen presenting cells (APCs) interact with the mother's microbial antigens and return to fetal peripheral lymphoid organs. The human fetus harbors large numbers of peripheral regulatory T cells (Tregs) with immunosuppressive activity (Takahata et al., 2004). Fetal Tregs can be retrieved also from umbilical cord blood offering a promising perspective for transplantation medicine, while newborns have been shown to have higher proportions of thymically derived Tregs than adults (Rabe et al., 2014). The fetal Tregs are preventing undesirable alloreactivity to maternal derivatives during the pregnancy (Takahata et al., 2004). As a result, the fetus develops prenatal tolerance to the mother's microbiome and regards it as “safe” during postnatal encounters with these bacteria. There is experimental and clinical evidence for development of fetal antigen-specific tolerance: human fetal Tregs become functionally suppressive after stimulation with maternal alloantigens and persist at least until early adulthood (Mold et al., 2008). If and to what extent early tolerance of the fetus toward the oral bacterial species is key to the colonization of the mouth and gastro-intestinal tract of the newborn and its impact in health development in the earliest phases of life needs to be investigated.

## Acquiring the oral microbiome

Vertical transmission from mother to child starts at birth. The delivery mode (vaginal or Caesarian) will to a large extent determine which microorganisms—vagina or skin-derived—will be encountered first by the newborn (Dominguez-Bello et al., 2010). This affects the diversity of the oral microbiome: vaginally born infants showed higher taxonomic diversity at 3 months of age (Lif Holgerson et al., 2011). Interestingly, the birth mode may have a lasting impact as infants born with Caesarian section acquired *Streptococcus mutans* almost 1 year earlier (at 17.1 months of age) than vaginally born infants (28.8 months) (Li et al., 2005).

The method of feeding (breast-feeding or infant formula) affects the infant's microbiome as well. Breast-fed infants of 3 months of age carried oral lactobacilli with antimicrobial properties not found in formula-fed infants (Holgerson et al., 2013; Romani Vestman et al., 2013). In addition to this vertical transmission mechanism, horizontal transmission of oral microbiota, e.g., among siblings and other people sharing the same environment, contributes to oral microbiome diversity (Baca et al., 2012; Stahringer et al., 2012).

Once established, the microbiome should be sustained. Below we summarize our perspective on this process by dividing it into host-derived and microbe-derived microbiome maintenance factors. Since microbial factors have been extensively addressed elsewhere (Kuramitsu et al., 2007; Wright et al., 2013), we focus here mainly on the role of host factors.

## Host-derived microbiome maintenance factors

The interactions between the microbiome and the host are bidirectional. In the absence of timely and adequate stimulation by the microbiome, germ-free mice show extensive deficiencies in intestinal immune system development, with reduced lymphoid tissue and fewer lymphocytes (Macpherson and Harris, 2004). In humans, early-life exposure to microbiota is protective for immune-mediated diseases such as asthma and inflammatory bowel disease (Olszak et al., 2012). The interplay between the microbiome and innate and adaptive immunity is site specific. The skin microbiome of mice is largely unaffected by genetic manipulation of innate or adaptive immunity, while mucosal sites—oral and gut microbiomes—are shaped by innate and adaptive immune responses (Scholz et al., 2014).

The human immune system develops in a continuing dialog with the commensal microbial populations. One communication route between microbiota and host is via the host pattern recognition receptors (PRRs), with the Toll-like receptor (TLR) family playing a key role. In the gut, ligands binding to TLR-2 can favor Treg expansion (Sutmuller et al., 2006), while TLR-9-mediated recognition of DNA from gut flora is essential for effector T-cells to overcome Treg inhibition and mount an immune response (Hall et al., 2008). Cells of the oral mucosa (keratinocytes, macrophages, dendritic cells (DCs), polymorphonuclear leukocytes and natural killer cells) express most of the TLRs (Feller et al., 2013), while altered expression patterns of TLRs have been found in oral pathology (Janardhanam et al., 2012).

Despite dense bacterial colonization, acute infections are rare in the oral mucosa, suggesting that this site is predominantly tolerogenic (Novak et al., 2008). Mucosal DCs are the arbiters of mucosal tolerance: on the one hand they are capable of mounting an effective defense against harmful pathogens, while on the other hand they inhibit immune reactions against antigens derived from commensal bacteria, preserving the health benefits acquired through thousands of years of co-evolution. DCs in the oral mucosal epithelium are of the Langerhans cell subtype and they employ complex regulatory mechanisms: deletion of T-cells via apoptosis, functional inactivation of T-cells, inhibition by co-inhibitory receptors, but also the development of antigen specific Tregs (Novak et al., 2008). Expression of LPS receptor CD14, TLR2, and TLR4 by DCs in the non-inflamed oral mucosa is crucial for the induction of tolerance. One of the mechanisms involved is the induction of Tregs characterized by interleukin (IL)-10 and transforming growth factor (TGF)-β secretion (Allam et al., 2008). In the gut, resident DCs are programmed by intestinal epithelial cells to suppress inflammation and promote immunological tolerance; in the case of a pathogen attack, “non-educated” DCs are recruited to initiate inflammation and a protective immune response against the invader (Iliev et al., 2007). A similar mechanism could also account for the oral mucosal tolerance. Indeed, a significant infiltration of pro-inflammatory plasmacytoid DCs is found in oral inflammation (Santoro et al., 2005).

Besides the PRRs, the host may also use chemical sensing to monitor microbial activity. Well known is epithelial signaling of short chain fatty acids involving dedicated G-coupled receptors (GPR41, GPR43) on gut epithelial cells (Layden et al., 2013). Recent studies suggest a direct link between secreted bacterial products and chemosensory activation mechanisms for mucosal clearance (Lee et al., 2014). Oral mucosa, but also airway epithelial cells and airway smooth muscle cells express bitter taste receptors (T2Rs) (Prince, 2012). The T2R38 receptor is activated directly by acyl-homoserine lactone (AHL) quorum sensing molecules produced by *Pseudomonas aeruginosa* and other Gram-negative bacteria and thus provide a mechanism for chemical sensing of bacterial colonization (Lee et al., 2012). Genetic differences in the T2R38 receptor, conferring an increased ability to perceive the bitter-tasting phenyl-thiocarbamide (supertaster phenotype), have been shown to underlie differences in the ability to signal presence and subsequent clearance of *P.aeruginosa* biofilm by respiratory epithelial cells (Lee et al., 2012). Intriguingly, specific alleles of the T2R38 bitter taste receptor have also been associated with a reduced risk for caries (Wendell et al., 2010). While this association was significant for the primary dentition, no significant associations could be identified for the mixed or permanent dentition groups. In addition, decreased taste sensitivity for another bitter-tasting chemical, 6-n-propylthiouracil, was associated with increased risk for dental caries and higher mutans streptococci counts (Shetty et al., 2014). Although the ability to taste bitter components may influence dietary habits, it is tempting to suggest a mechanistic relationship between bitter taste and antimicrobial defense also in the oral cavity.

An important oral immunity factor is delivered *via* saliva and gingival crevicular fluid—the secretory immunoglobulin A (S-IgA). These antibodies limit and control microbial adhesion and colonization (Feller et al., 2013). IgA proteases that neutralize S-IgA are known virulence factors of human pathogens such as *Neisseria meningitidis* and *Streptococcus pneumoniae*. However, also commensal streptococci such as *Streptococcus mitis 1, Streptococcus oralis* and *Streptococcus sanguinis* are able to produce IgA proteases (Kilian et al., 1996). These commensal oral streptococci are primary colonizers and the major species colonizing infants and mucosal sites in adults (Marsh et al., 2009). Their ability to circumvent S-IgA guarantees survival in the oral cavity and underlines their long-lasting symbiotic co-evolution with the human host.

Salivary glycoproteins contain glycans that may act as decoys to prevent pathogens from adhering to epithelial cells, thereby influencing a healthy microbial homeostasis. This was shown *in vitro* on inhibition of adhesion of the fungus *Candida albicans* to epithelial cells (Everest-Dass et al., 2012). Other salivary proteins that influence the oral microbiome include lactoferrin, agglutinins, lysozyme, peroxidase, statherin, histatins, defensins, and mucins (for extensive review see Dodds et al., 2005). For instance, histatin 5 has candicidal activity and its concentration is reduced in saliva of elderly and HIV patients (Khan et al., 2013)—two populations prone to candidiasis. Salivary flow rate as well as composition thus play key role in maintaining a healthy oral microbiome.

The impact of the immune system on oral health is most obvious once it becomes dysfunctional, as in hematopoietic stem cell transplant patients who have received immunosuppressive therapy. In these patients, the mucosal barrier is often damaged leading to severe mucositis with life-threatening viral and fungal infections (Petti et al., 2013), as well as oral infections by non-oral species (Soga et al., 2011; Diaz et al., 2013).

## Microbe-derived microbiome maintenance factors

Co-evolution of the microbiome with the host has resulted in host-associated microbial communities that are equipped with mechanisms that allow them to prevent colonization and establishment of foreign microbes, so called “colonization resistance” (He et al., 2013). An extensive review on interspecies interactions within oral communities has been provided by Kuramitsu and colleagues. The authors distinguished five types of interaction between oral bacteria: competition for nutrients, synergy, antagonism, neutralization of virulence factors and interference in signaling mechanisms (Kuramitsu et al., 2007). Integrity of the microbial community is maintained by specific inter-microbial adhesion, cell signaling through cell-to-cell contact, metabolic interactions and quorum sensing (Wright et al., 2013). Recently a social structure in the murine oral community was reported where a concerted action by “sensor,” “mediator” and “killer” bacteria in the community formed a pathway that prevented colonization by the non-oral species *Escherichia coli* (He et al., 2013).

Besides bacterial inter-species communication, also inter-kingdom communication plays a role in oral microbial ecosystem (Morales and Hogan, 2010; Jarosz et al., 2011). Bacteria produce a range of signaling molecules that affect *C. albicans* biofilm formation or morphogenesis, while *C. albicans* metabolites are known to influence bacterial growth (Wright et al., 2013). Such interactions, as well as interkingdom adhesion events, are also likely with the other members of the oral mycobiome (Krom et al., 2014).

## Future research directions

A stable ecosystem is the result of complex interactions between all members of the system (Jenkinson, 2011). The entire oral microbiome of an individual, in addition to bacteria also fungi, viruses, Archaea and protozoa, has so far not been approached as an entity. The current knowledge is based on studying individual community components without the complexity of their mutual interactions. The first article on assessment of both, fungal and bacterial profiles in the same oral samples has just been published (Mukherjee et al., 2014), although focusing on diseased individuals, infected with HIV.

Microbiology of oral health has been neglected for decades. With the advent of health-related microbiome research grants such as HMP, an important turning point has been set, with a paradigm shift from disease as a starting point toward health as main interest. Since our current knowledge on the mechanisms of oral microbiome stability is only revealing the tip of the iceberg any initiatives similar to NIH HMP need to be encouraged. The Dutch Top Institute of Food and Nutrition (TIFN) (www.tifn.nl) has established public-private partnership that aims to identify the biological processes in the oral ecosystem responsible for maintaining oral health and to develop *in vitro* and *in vivo* technologies for the development of novel preventative strategies and to evaluate their efficacy. The project is based on the hypothesis that oral health reflects the ability of the oral ecosystem to adapt to and counteract perturbing stresses, where the oral ecosystem is defined as the oral microbiota, the saliva and host (mucosal) immunity.

The introduction of novel sequencing technologies has led to a significant advance in our knowledge of the oral microbiome in its broadest sense, and has revealed a stable commensal population, suggesting symbiotic beneficial relationship. However, the underlying principles of the beneficial interactions between host and the oral microbiota urgently require attention. Intriguing questions relate to the acquisition of the oral microbiota in early life, with a possible involvement of pre-natal tolerance development toward the oro-pharyngeal microbiota of the mother. Why do placentas in healthy pregnancies harbor microbiota at all and why does this microbiome resemble the oral communities? Is the fetus already seeded with maternal microbes *in utero*, as has been proposed recently (Aagaard et al., 2014)? How are these microbiota transported to placenta? Is this transportation selective? Do fetal APCs acquire antigens from placental tissues? If our views on fetal tolerance training by the placental microbiome are confirmed, how is the immune-modulation of the fetus guided by the placental microbiome? Are then vital bacteria involved in recognition by fetal immune system, or just their DNA fragments?

Once the microbiome is established, sustaining it at health becomes an issue. Colonization resistance mechanisms are just becoming apparent and more research needs to be performed using complex model systems and longitudinal clinical studies. Extremely exciting is the overlap between taste and chemical sensing of bacteria in the airways with possible similar mechanisms operating in the mouth. In addition to modulating taste, does the oral microbiota dictate the feeling of hunger or craving for specific dietary habits? Such interactions have been suggested recently where ingestion of bacterial LPS was shown to inhibit taste responses to sucrose in mice (Zhu et al., 2014). Substantial research has been done in the past few years, while one thing is certain—much more research needs to be done in the near future, leading to exciting discoveries directed to maintenance of oral and overall health in an ever-changing population.

### Conflict of interest statement

The authors declare that the research was conducted in the absence of any commercial or financial relationships that could be construed as a potential conflict of interest.
